# Supplementary figures and images for: Gender biases and hate speech: Promoters and targets in the Argentinean political context
Source: PLoS One. 2025 Jan 24;20(1):e0317001. doi: 10.1371/journal.pone.0317001 (PMC11760012; doi:10.1371/journal.pone.0317001)

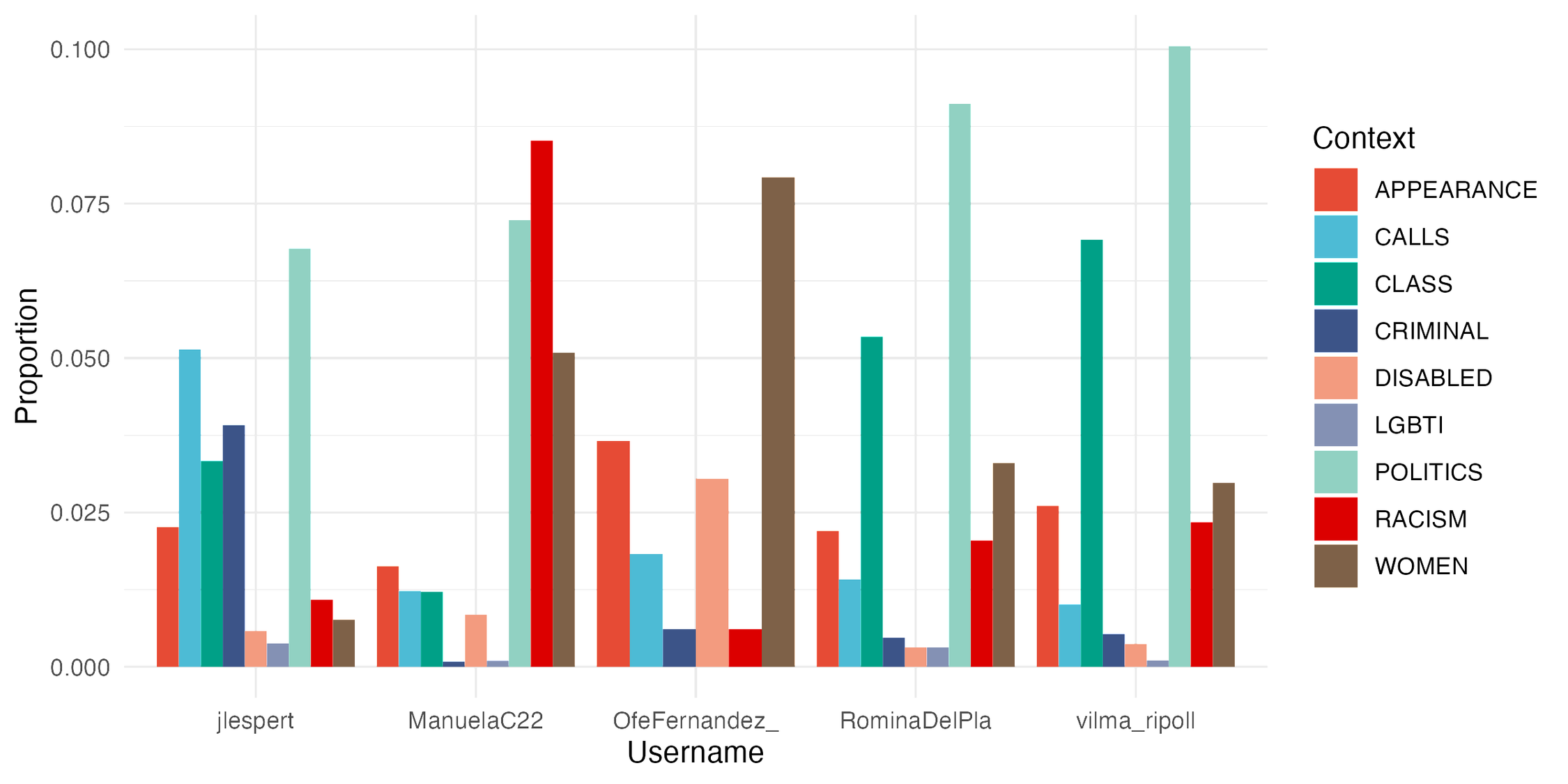

Supplement: S1 Fig — (TIF) [file pone.0317001.s001.tif]

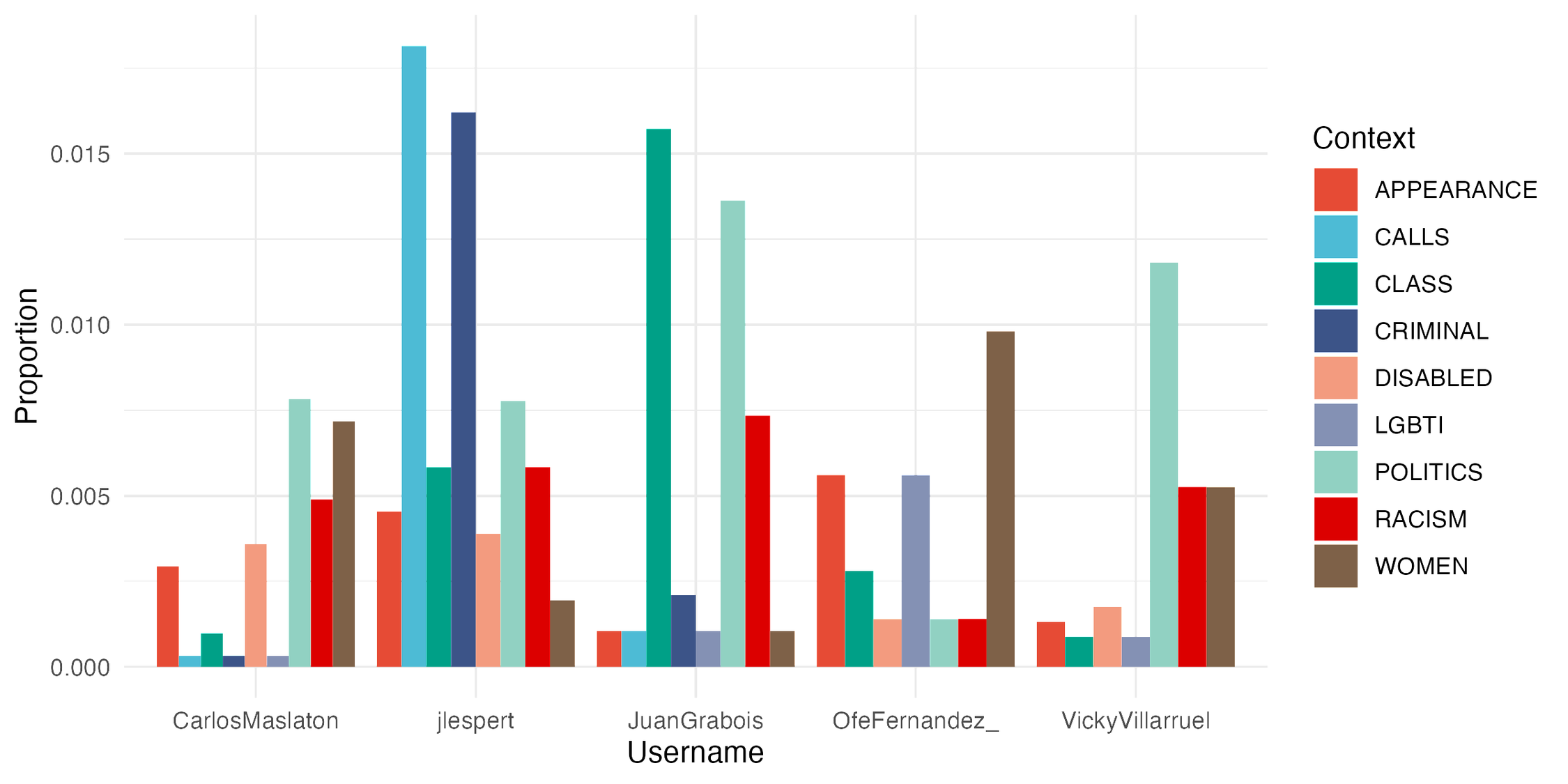

Supplement: S2 Fig — (TIF) [file pone.0317001.s002.tif]

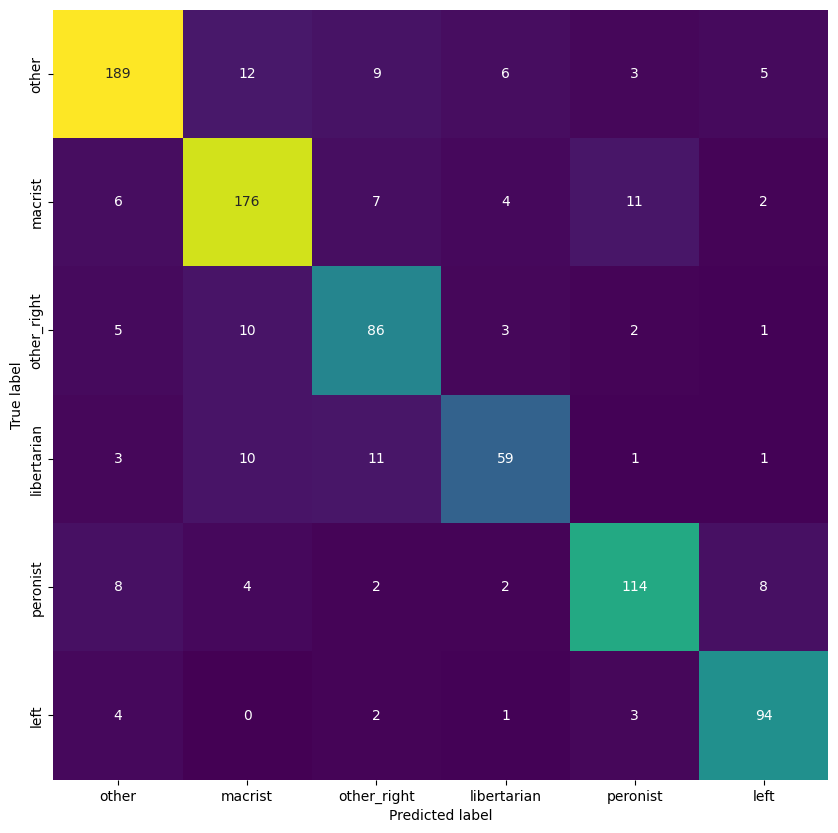

Supplement: S3 Fig — (TIF) [file pone.0317001.s003.tif]
