# Supplementary material for: Gender biases and hate speech: Promoters and targets in the Argentinean political context
Source: PLoS One. 2025 Jan 24;20(1):e0317001. doi: 10.1371/journal.pone.0317001 (PMC11760012; doi:10.1371/journal.pone.0317001)
Supplement: Attachment — Submitted filename: R2R.docx [file pone.0317001.s008.docx]

Journal Requirements:

1. Please ensure that your manuscript meets PLOS ONE's style requirements, including those for file naming. The PLOS ONE style templates can be found at

https://journals.plos.org/plosone/s/file?id=wjVg/PLOSOne_formatting_sample_main_body.pdf and

<https://journals.plos.org/plosone/s/file?id=ba62/PLOSOne_formatting_sample_title_authors_affiliations.pdf>

**Answer:**

We have modified the format to adapt it to PLOS ONE style.

Please note that PLOS ONE has specific guidelines on code sharing for submissions in which author-generated code underpins the findings in the manuscript. In these cases, all author-generated code must be made available without restrictions upon publication of the work. Please review our guidelines at https://journals.plos.org/plosone/s/materials-and-software-sharing#loc-sharing-code and ensure that your code is shared in a way that follows best practice and facilitates reproducibility and reuse.

**Answer:**

All the code is available at a github repository (<https://github.com/ldmnch/twitter_hate_speech>) which will be made public upon acceptance.

Please remove your figures from within your manuscript file, leaving only the individual TIFF/EPS image files, uploaded separately. These will be automatically included in the reviewers’ PDF.

**Answer:**

All figures were removed and uploaded as TIFF

Please upload a copy of Table S1 to S5 and Figures S1 to S6 to which you refer in your text. Please amend the file type to 'Supporting Information'. If the Supplementary file is no longer to be included as part of the submission please remove all reference to it within the text.

**Answer:**

Sorry for the confusion. We have revised all the supplementary material references in the texts and upload it accordingly

Please include captions for your Supporting Information files at the end of your manuscript, and update any in-text citations to match accordingly. Please see our Supporting Information guidelines for more information: http://journals.plos.org/plosone/s/supporting-information.

**Answer:**

All captions were added

**Reviewer #1:**

While the paper provides a comprehensive review of existing literature, it lacks a clear theoretical framework to guide the analysis. Incorporating theoretical perspectives from communication studies, sociology, or critical discourse analysis could enhance the paper's theoretical depth and analytical rigor. Moreover, the paper briefly mentions methodological approaches such as text mining, machine learning, and topic modeling but lacks detailed descriptions of the methodologies employed in the studies reviewed. Providing more information about the specific methods used in hate speech detection and analysis would help readers evaluate the validity and reliability of the findings.

**Answer:**

We thank the reviewer for this comment. We agree that an extended theoretical framework was needed. For this, we expanded the introduction and added a section 2. Theoretical Framework and divided the introduction to add a section 3. Related work.

While the paper acknowledges the contextual nature of hate speech, it could benefit from a more in-depth exploration of regional variations, particularly in the Latin American context. Providing case studies or examples of hate speech dynamics in specific regions would enrich the analysis and facilitate a more nuanced understanding of the phenomenon.

**Answer:**

We agree that a better contextualization of hate-speech in Latin America was needed. We extended the introduction to add this context.

Hate speech detection and analysis raise ethical concerns related to privacy, bias, and potential harm to vulnerable communities. The paper could benefit from a discussion of ethical considerations inherent in hate speech research and how researchers address these concerns in their methodologies and practices.

**Answer:**

We agree that automatic hate speech raises moral concerns that need to be acknowledge. Following Reviewer #2 comments, we added a dedicated section 4.4. on the limitations of our methodology.

**Reviewer #2:**

A “Related work” section is completely missing and without it it’s very difficult to compare this work with state of the art methods and consequently the impact of your scientific contributions. Please integrate such section in order to report which results and findings have been obtained in other existing works and how eventually your work differs from existing ones.

**Answer:**

We agree with the reviewer that adding a Related work section can help to better contextualise our contribution. We added this section to the current manuscript.

The use of advanced commercial or open-source large language models (LLMs) (e.g., ChatGPT, Mixtral, etc.) as zero-shot classifiers for automatic classification of aspects of interest (sentiment, hate speech, etc.) should be considered in your work as a better alternative to overcome some of the limitations of the ad-hoc models used in this study. In particular, such LLMs are generally very capable of analyzing text correctly and contextualizing input text, even in cases where content originates from social media (e.g., slang, emojis, etc.). In addition, such tools are able to properly interpret a text by determining if some hate speech is against someone specific, not just by considering if in the text there are specific keywords or user mentions that coud confuse a basic NLP method based on traditional machine learning. Therefore, the use of such tools could provide more accurate results, ultimately improving the overall quality of your findings.

**Answer:**

We thank the reviewer for this suggestion. Indeed, using a LLM allowed us to further refine the results. Although our experiments showed that pysentimiento (Pérez JM, Giudici JC, Luque F. pysentimiento: A Python Toolkit for Sentiment Analysis and SocialNLP tasks. arXiv; 2021) has a better performance to detect hate speech for this very specific linguistic context, our qualitative analysis also showed that some of the hate speech detected, although it was real hate speech, was not always targeting the mentioned candidate. In some cases, for example, it was hate speech that was supporting the original hateful message of a candidate. To complement the hate-speech detection and the qualitative analysis, we used chat GPT to fine tune our results with the prediction of the directionality of the hate speech (to detect if the candidates where or not the target of the message). These results were added at the end of section 5.3. together with the new figures 4 and 5.

To enhance content organization and clarity, I recommend dedicating a separate section to discussing potential limitations of the methodology applied in this study. This approach will enable readers to focus more easily on the research design and methods used, while also reflecting on aspects related to the generalizability of the findings.

**Answer:**

We agree that a dedicated section for the limitations simplifies the reading of the methods. We compiled all the discussion on limitations on the new section 4.4 and the end of Data & Methods.

Unable to find in the article some referenced tables and/or figures (e.g. Table S5, Figure S6, etc.).

**Answer:**

Sorry for this omission, we submitted the supplementary materials in the current version.
