# Supplementary material for: Gender biases and hate speech: Promoters and targets in the Argentinean political context
Source: PLoS One. 2025 Jan 24;20(1):e0317001. doi: 10.1371/journal.pone.0317001 (PMC11760012; doi:10.1371/journal.pone.0317001)
Supplement: Attachment — Submitted filename: R2R_2.docx [file pone.0317001.s009.docx]

Editorial team:

1. However, our editorial team has noted the following text in the manuscript:

"Also, in our random sample of 15 cases we retrieved five tweets from the same user —'TurcoWturco'— mentioning a conspiracy theory of a cloned sister of Fernandez, which reflects on a systematic type of violence, rather than spontaneous social media comments. This user repeated the same tweet several times (each time mentioning different users), which could indicate that it is a troll."

It is not clear that naming the specific user is necessary for the accuracy or replicability of the study. We would therefore request that you please revise the submission to remove the user name, or include a clear scientific rationale for its inclusion.

**Answer:**

We thank the editorial team for this comment. Indeed, highlighting the name of a single user is unnecessary and detrimental for the article. We therefore removed the user name.

**Reviewer #2:**

I appreciate the fact that the authors have tried to include the suggestions proposed by reviewers to improve the quality of the paper. After the revisions, the paper is more comprehensible, and the scientific contribution it provides is now at an appropriate level for publication. However, after reading the revised paper I have some other suggestions in order to improve the work. The new section “Related Work,” although it includes a substantial amount of relevant studies, does not clearly specify how the proposed work improves upon the state of the art. Neither in the “Related Work” section nor in the “Introduction” is there a discussion of the current limitations of previous studies on hate speech in the context of Argentina or South America, and how the proposed study addresses these gaps. For example, has gender bias in hate speech ever been studied in South America or Argentina? If so, what has been done, and what are the limitations of such studies? Explicitly addressing these questions could help clarify the knowledge gap in the current literature that your work seeks to fill. Although not mandatory (I am not asking an additional review), I suggest the authors to also cover these points in the final version of the article, to improve readability and motivations of their work.

**Answer:**

We thank the reviewer for this comment. We agree that explicitly addressing these questions clarify the knowledge gap in literature and the relevance for our work in the field. To address this, we extended a paragraph in the introduction, to include the research gap before the research questions.
